# Curcumin Inhibits Proliferation of Epstein–Barr Virus-Associated Human Nasopharyngeal Carcinoma Cells by Inhibiting EBV Nuclear Antigen 1 Expression

**DOI:** 10.1155/2019/8592921

**Published:** 2019-10-07

**Authors:** Limei Liu, Jiaomin Yang, Wuguang Ji, Chao Wang

**Affiliations:** ^1^Department of Keratonosus, Weifang Eye Hospital, Weifang 261053, Shandong, China; ^2^Department of Laboratory Medicine, WeifangYidu Central Hospital, Qingzhou 262500, Shandong, China; ^3^Department of Vascular Surgery, Weifang Peoples's Hospital, Weifang 261053, Shandong, China; ^4^Department of Pathogen Biology, Weifang Medical University, Weifang 261053, Shandong, China

## Abstract

This investigation aims to study the effect of curcumin on the proliferation, cycle arrest, and apoptosis of Epstein–Barr virus- (EBV-) positive nasopharyngeal carcinoma (NPC) cells. EBV^+^ NPC cells were subjected to curcumin treatment. The cell viability was evaluated with the CCK-8. Cell cycle and apoptosis were analyzed by flow cytometry analysis. Expression (protein and mRNA) levels were detected with western blotting and quantitative real-time PCR, respectively. Curcumin efficiently reduced the viability of EBV^+^ NPC cells. Curcumin induced the cycle arrest of the HONE1 and HK1-EBV cells positive for EBV. Moreover, curcumin treatment promoted the NPC cell apoptosis, via the mitochondria- and death receptor-mediated pathways. Furthermore, curcumin decreased the expression of EBNA1 in the HONE1 and HK1-EBV cells and inhibited the transcriptional level of EBNA1 in the HeLa cells. Curcumin induced EBNA1 degradation via the proteasome-ubiquitin pathway. In addition, curcumin inhibited the proliferation of HONE1 and HK1-EBV cells positive for EBV, probably by decreasing the expression level of EBNA1. In both the HONE1 and HK1-EBV cells, curcumin inhibited the EBV latent and lytic replication. Curcumin could reduce the EBNA1 expression and exert antitumor effects against NPC *in vitro*.

## 1. Introduction

Epstein–Barr virus (EBV), a well-known common *γ*-herpes virus, induces asymptomatic infection in approximately 95% adults throughout the world [1]. The EBV infection has been regarded as the high-risk factor related to more than 1% cancer cases [2]. Moreover, the infection of EBV has also been implied in various proliferative diseases associated with epithelial or lymphoid cells in human beings, such as the gastric carcinoma, nasopharyngeal carcinoma (NPC), Burkitt's lymphoma, and Hodgkin's lymphoma [3]. As a malignant tumor, NPC mainly originates from the nasopharynx epithelium, which is endemic in South China and Southeast Asia, and the disease incidence is 20–50 cases per 100,000 people [4, 5]. In NPC, EBV infection has type II latency mechanism [6], which is featured by several noncoding RNAs, latent membrane proteins (LMP1, LMP2A, and LMP2B), and Epstein–Barr nuclear antigen 1 (EBNA1) [7]. Due to the special viral antigen expression, NPC has become an attractive target for clinical therapies.

The essential viral protein EBNA1 is expressed in tumors associated with EBV, during the viral latency processes (but not for latency 0) [8]. EBNA1 plays vital roles in the maintenance, replication, and segregation of latent episomes [9]. Moreover, EBNA1 could interact with the cellular and viral promoters, regulating the gene transcription level [9]. A recent study has shown that EBNA1 has oncogenic activity, and this pleiotropic protein regulates its own synthesis to escape from the host cells exerting immune functions [10]. Furthermore, EBNA1 has also been shown to be able to decrease p53 accumulation in the epithelial cells infected with EBV, which might disrupt the antitumor function of these cells [11]. Although expressed in malignancies associated with EBV, the EBNA1 expression would obey various regulating modes, which depends on the types of host cells. Under most circumstances, the EBNA1 transcription would be promoted by the Q promoter (Qp) (in both type I or II latency) [12]. For the NPC cells (type II infection), Qp could mediate EBNA1 transcription [13]. Furthermore, it has been suggested that EBNA1 would be necessary the tumor cell proliferation [14]. EBNA1 is recognized as an attractive target in various therapeutic strategies in clinics.

Curcumin (Cur) is a polyphenol extracted from *Curcuma longa*, which has been extensively investigated due to its modulating effects of signaling pathways related to proliferation and invasion of cells, as well as angiogenesis [15, 16]. Curcumin can exert pharmacological functions, such as antimicrobial and anti-inflammation functions [17–19]. Moreover, curcumin is able to block the cancer growth of various types, including prostate cancer, NPC, and other cancers [20, 21]. Curcumin could reduce human breast cancer cell (MDA-MB-231) proliferation by decreasing the expression level of Bcl-2 and upregulating the Bax, PARP, and cleaved caspase 3 expression levels [22]. A previous study has shown that curcumin could sensitize the NPC cells to radiation via modulating the noncoding RNA levels, Jab1/CSN5, and/or ROS generation [23]. Furthermore, curcumin is shown to cause NPC cell apoptosis through both the mitochondria-dependent and death receptor-mediated pathways [24].

Our previous study had shown that heat shock protein 90 (Hsp90) inhibitors could block the proliferation of malignant cells infected with EBV, both *in vivo* and *in vitro* [25]. It has also been suggested that triptolide treatment could inhibit EBV^+^ B lymphocyte proliferation through inhibiting LMP1 [26]. Furthermore, we have also shown that berberine shows the antitumor activity against NPC through the EBNA 1-dependent mechanism [13]. Furthermore, triptolide could decrease the human telomerase reverse transcriptase stability in BC-3 and BCBL-1 cells [27].

In this study, effects of curcumin on cell proliferation, cellular apoptosis, and cycle arrest of EBV^+^ NPC cells were investigated. Moreover, mechanisms through which curcumin inhibited NPC were also analyzed.

## 2. Materials and Methods

### 2.1. Study Cells

EBV^+^ NPC cells (i.e., the HONE1 and HK1-EBV cells) were kind gifts from Prof. SaiWahTsao (University of Hong Kong, Hong Kong, China). These cells were cultured with the RPMI-1640 medium, which contained G418 (400 ng/ml) and 10% fetal bovine serum (Gibco-BRL). HeLa cells were cultured in the DMEM medium, which also contained 10% fetal bovine serum. These cells were cultured at 37°C with 5% CO_2_.

### 2.2. Cellular Viability Assessment

Cellular viability was analyzed with the Cell Counting Kit-8 (CCK-8) (Dojindo Laboratories). The cells were planted onto the 96-well plate (1 × 10^4^ cells/100 *μ*l), which were incubated with DMEM. These cells were incubated with 0.006% DMSO (vehicle control) or increasing concentrations of curcumin (Sigma-Aldrich, Shanghai, China), for 24 and 48 h, respectively. Then, 10 *μ*l CCK-8 was added to incubate the cells in dark at 37°C for 1 h. The OD_450_ value was detected with an EL × 800 microimmuno analyzer (BioTek Instruments, Inc.).

### 2.3. Flow Cytometry

Annexin V-FITC/PI apoptosis detection kit (Multisciences) was used to analyze apoptosis. HK1-EBV and HONE1 cells were incubated with 0.006% DMSO (vehicle control) or curcumin (5 *μ*M and 10 *μ*M). After 24 h, cells were resuspended in the binding buffer (500 *μ*l). The cells were incubated with Annexin V-FITC (5 *μ*l) and PI (10 *μ*l). After that, cells were analyzed with EPICS Altra II (Beckman Coulter).

### 2.4. Transfection

The plasmids of pSG-5-EBNA1 and pGL3.0-Qp were synthesized by the Neuron Biotech Corporation (Shanghai, China). Transient cellular transfection was performed with X-tremeGENE HP DNA Transfection Reagent (Roche). After 4 h of transfection, 0.006% DMSO (control) or curcumin was added and incubated for 44 h.

### 2.5. Dual Luciferase Reporter Assay

After cotransfection with 100 ng pGL3.0-Qp and 5 ng pRL-TK for 4 h, HeLa cells were incubated with 0.006% DMSO or curcumin (5 or 10 *μ*Μ). After 24 h, the total protein was harvested with the 1Χ lysis buffer (Promega, Madison, WI, USA). Luciferase activity was measured by the GLO-MAX 20/20 system (Promega, Madison, WI, USA). *Renilla* was as internal control reference.

### 2.6. Quantitative Real-Time PCR

The total RNA was extracted with TRIzol (Invitrogen, Grand Island, NY, USA). First-strand cDNA was synthesized with the Reverse Transcription kit (Takara, Tokyo, Japan). Quantitative real-time PCR was conducted on the CFX96 real-time PCR detection system, with the SYBR Premix Ex Taq kit (Takara, Tokyo, Japan). Primer sequences were as follows: EBNA1, forward 5′-GGTCGTGGACGTGGAGAAAA-3′ and reverse 5′-GGTGGAGACCCGGATGATG-3′; and GAPDH, forward 5′-ACATCGCTCAGACACCATG-3′ and reverse 5′-TGTAGTTGAGGTCAATGAAGGG-3′. The reaction conditions were set as follows: 95°C for 30 s; 95°C for 10 s, 62°C for 10 s, and 72°C for 15 s, for a total of 45 cycles. The house-keeping gene GAPDH was as internal reference.

### 2.7. Western Blotting

Cells were lysed with the RIPA buffer (Beyotime, Shanghai, China), containing 0.5% cocktail protease inhibitor (Roche) and 0.5 mM PMS, on a microscraper. After sonication for 15 s, the extract was centrifuged (12,000 ×*g*) for 15 min. Protein concentration was determined with the BCA method (standard sample: bovine serum albumin). Proteins were separated with 10% SDS-PAGE and transferred onto the PVDF membrane. After blocking with 5% skim milk in TBST for 1 h, the PVDF membrane was cultured with the anti-caspase-3 (Cat. #9668; CST, CA, USA), anti-mouse anti-EBNA1 (Cat. #sc81581; Santa Cruz, Santa Cruz, CA, USA), anti-caspase-9 (Cat. #A2636l; ABclonal, Boston, UK), anti-cleaved caspase-3 (Cat. #9664; CST), anti-p53 (Cat. #sc-126; Santa Cruz), anti-cleaved PARP-1 (Cat. No. sc-56, 196; Santa Cruz), anti-GAPDH (Cat. #10494-1-AP; Proteintech, Wuhan, Hubei, China), anti-Fas (Cat. #8023; CST), and anti-FasL (Cat. #4273; CST), respectively, at 4°C overnight. Then, the membrane was washed with TBST and then cultured with the HRP-conjugated anti-rabbit IgG (Wuhan Keri Technology) and anti-mouse IgG (Wuhan Keri Technology) secondary antibodies, respectively, for 1 h. Immunoreactivity was detected with ECL. Image *J* software was used to process images.

### 2.8. Statistical Analysis

Data have been presented as the mean ± SD. Comparison was conducted with Student's *t*-test, with the GraphPad Prism (GraphPad Software, La Jolla, California, USA). For statistical analysis, *P* < 0.05 was considered as statistically significant.

## 3. Results

### 3.1. Curcumin Suppresses Proliferation of NPC Cells Positive for EBV

**Figure 1:**
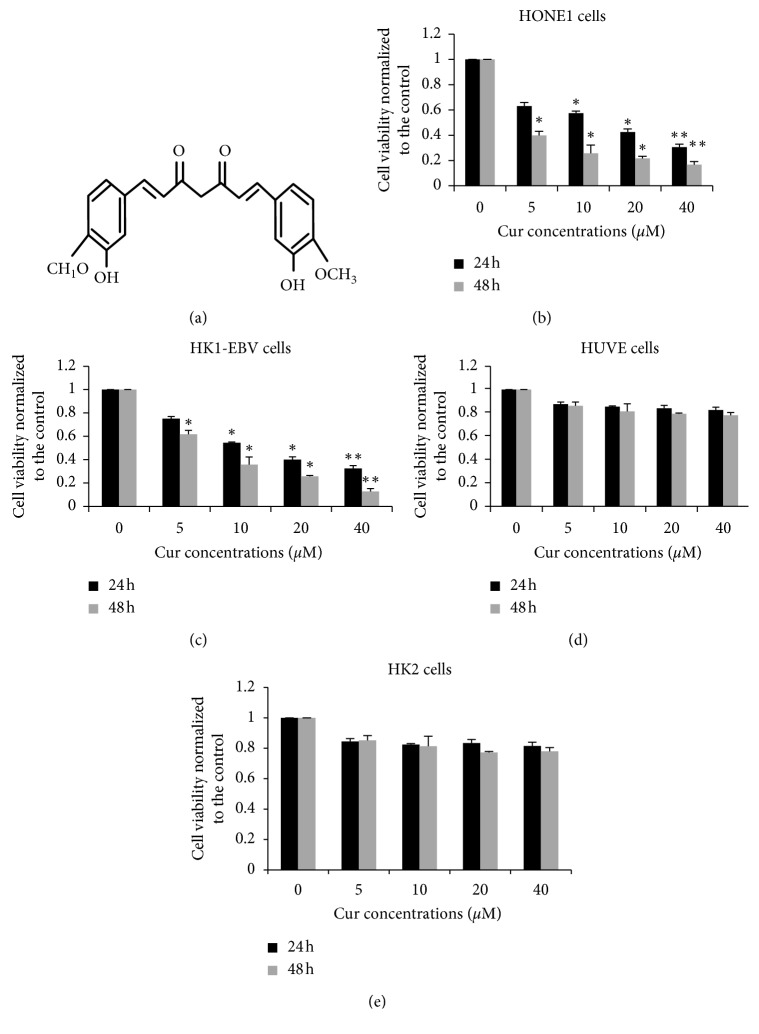
Curcumin suppresses proliferation of EBV-positive NPC cells. (a) Chemical structure of curcumin. HONE1 (b), HK1-EBV (c), HUVE (d), and HK2 (e) cells were treated with DMSO (vehicle control) or curcumin (5 *μ*M, 10 *μ*M, 20 *μ*M, or 40 *μ*M) for 24 h and 48 h, respectively. Cell proliferation was assessed using the CCK-8. Compared with the vehicle control (0.006% DMSO) group, ^*∗*^*P* < 0.05, ^*∗∗*^*P* < 0.01.

To investigate the effects of curcumin treatment on the cellular viability of NPC cells positive for EBV, HONE1, and HK1-EBV, cell proliferation was assessed with the CCK-8, after drug administration. As shown in Figure 1, curcumin reduced HONE1 cell viability, in not only a dose-dependent manner but also a time-dependent manner. Moreover, the decline in HONE1 cell viability ranged over 35%–90% after treatment with curcumin for 24 h and 48 h. For the HONE1 cells, the 50% inhibitory concentration (IC_50_) values were 12.4 *μ*M and 3.3 *μ*M for the 24 h and 48 h curcumin treatment, respectively. On the contrary, similar findings were obtained for the HK1-EBV cells subjected to the treatment series of curcumin. Moreover, after 24 h and 48 h curcumin treatment, the HK1-EBV cell viability was reduced from 22% to 81%. Furthermore, for the HK1-EBV cells, the IC_50_ values were 10.4 *μ*M and 7.2 *μ*M for the 24 h and 48 h curcumin treatment, respectively (Figure 1(c)). In addition, the concentration series of curcumin treatment slightly inhibited the proliferation of the HUVE and HK2 cells (Figures 1(d) and 1(e)). These findings demonstrate that curcumin could efficiently decrease the EBV^+^ NPC cell viabilities but not for the HUVE and HK2 cells.

### 3.2. Curcumin Leads to Cell Cycle Arrest in NPC Cells Positive for EBV

**Figure 2:**
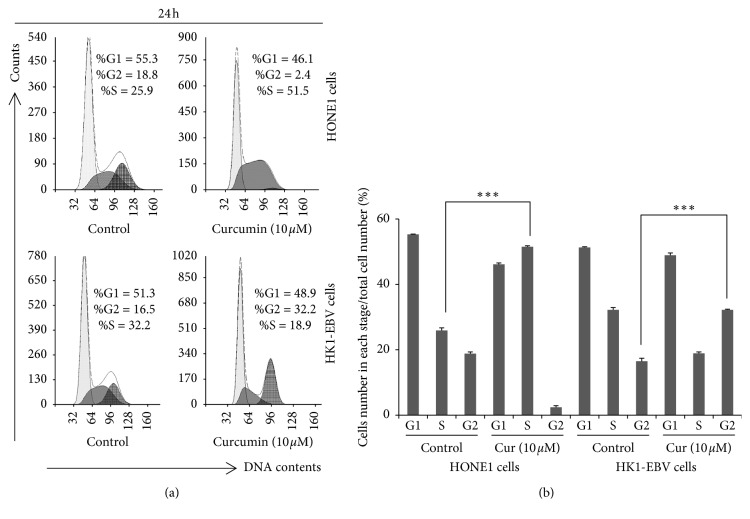
Curcumin induces cell cycle arrest in EBV-positive NPC cells. (a) Effect of curcumin on the cell cycle. HONE1 and HK1-EBV cells were treated with DMSO (vehicle control) or curcumin at indicated concentrations, for 24 h. Cell cycle was analyzed by flow cytometry. (b) Statistical analysis of the cell cycle. Compared with the vehicle control (0.006% DMSO) group, ^*∗*^*P* < 0.05, ^*∗∗*^*P* < 0.01.

To investigate the effects of curcumin on the cell cycle of NPC cells positive for EBV, flow cytometry was performed after drug treatment. The treatment of 10 *μ*M curcumin led to an arrest in the S phase in the HONE1 cells, with the fraction of S-phased cells increased by 25.6% (Figure 2). On the contrary, in the HK1-EBV cells, the curcumin treatment (10 *μ*M) led to a G2 arrest, with the cell fraction of G2-phase increased by 15.7%. These results reveal that curcumin could induce cell cycle arrest of NPC cells positive for EBV.

### 3.3. Curcumin Enhances NPC Cell Apoptosis via Mitochondria- and Death Receptor-Dependent Pathways

**Figure 3:**
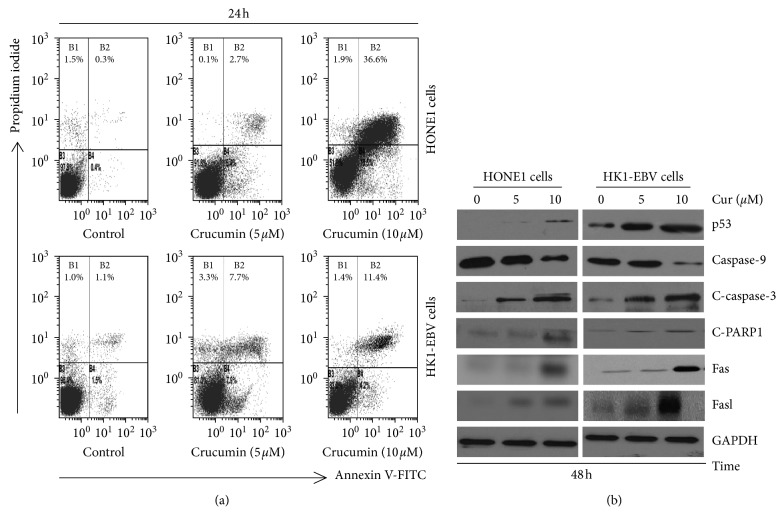
Curcumin induces cell apoptosis in EBV-positive NPC cells. (a) Effect of curcumin on cell apoptosis. HONE1 and HK1-EBV cells were treated with DMSO (vehicle control) or curcumin at indicated concentrations, for 24 h. Cellular apoptosis was analyzed by flow cytometry. (b) HONE1 and HK1-EBV cells were treated with DMSO (vehicle control) or curcumin at indicated concentrations, for 48 h, and the related protein expression levels were determined by western blot analysis.

Effect of curcumin treatment on NPC cell apoptosis was analyzed. As shown in Figure 3, the curcumin treatment enhanced apoptosis in the HK1-EBV and HONE1 cells, in a dose-dependent manner. Moreover, the apoptosis-related protein expression levels had been examined with the western blotting. Curcumin decreased the caspase-9 level, while increased the cleaved caspase-3, p53, cleaved PARP-1, Fas, and FasL expression levels, in the HK1-EBV and HONE1 cells (Figure 3). The findings suggest that curcumin could induce NPC cell apoptosis via both the mitochondria- and death receptor-dependent pathways.

### 3.4. Curcumin Downregulated EBNA1 Expression Levels in NPC Cells Positive for EBV

**Figure 4:**
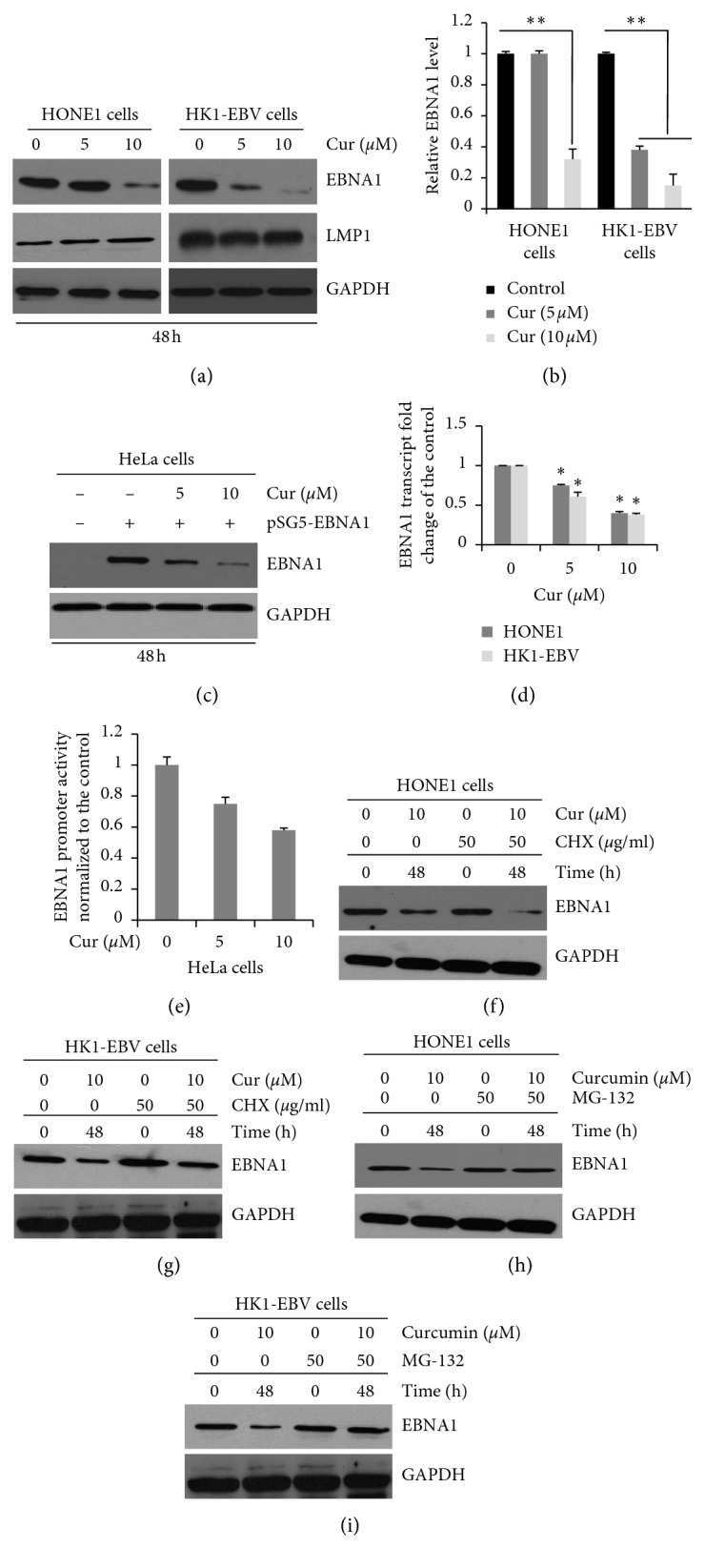
Effects of curcumin on transcription, expression, and half-time of EBNA1 in HONE1 and HK1-EBV cells. (a) HONE1 and HK1-EBV cells treated with DMSO (vehicle control) or curcumin (5 *μ*M and 10 *μ*M), for 48 h. (b) Statistical analysis of the expression levels of EBNA1 in the HONE1 and HK1-EBV cells. (c) HeLa cells transfected with pSG5 or pSG5-EBNA1, followed by the treatment of curcumin (5 *μ*M and 10 *μ*M) for 44 h (d) HONE1 and HK1-EBV cells treated with DMSO (vehicle control) or curcumin (5 *μ*M and 10 *μ*M), for 24 h (e) HeLa cells cotransfected with pRL-TK and pGL3.0-Qp, followed by the treatment with DMSO (vehicle control) or curcumin (5 *μ*M and 10 *μ*M). The activity of the promoter was determined by the Dual-Luciferase reporter assay. The HONE1 (f) and HK1-EBV (g) cells treated with DMSO (vehicle control) or curcumin (10 *μ*M), in the presence or absence of CHX (50 *μ*g/ml). The HONE1 (h) and HK1-EBV (i) cells treated with DMSO (vehicle control) or curcumin (10 *μ*M), in the presence or absence of MG-132 (50 *μ*g/ml). Transcription levels of EBNA1 were determined by quantitative real-time PCR. Western blot analysis was performed to detect the protein expression levels. Compared with the vehicle control (0.006% DMSO) group, ^*∗*^*P* < 0.05, ^*∗∗*^*P* < 0.01.

To investigate whether curcumin could alter the expression levels of EBNA1 in the NPC cells positive for EBV after drug administration, the HK1-EBV and HONE1 cells were detected with western blotting. EBNA1 levels in the HONE1 cells were not dramatically altered in the 5 *μ*M curcumin treatment group compared with the control group, which were downregulated to 29.8% for the 10 *μ*M curcumin treatment group (Figures 4(a) and 4(b)). Moreover, the EBNA1 expression levels in the HK1-EBV cells were decreased to 30.5% and 12.7% of the control group, after curcumin treatment (5 and 10 *μ*M). At the same time, another important protein expressed by EBV, LMP1, was also investigated. Our results showed that curcumin could not affect the expression of LMP1 in HONE1 and HK1-EBV cells.

To determine if curcumin specifically decreases the EBNA1 expression levels, HeLa cells were transfected with pSG5-EBNA1 for 4 h, which were then subjected to treatment with or without curcumin. In the pSG5-EBNA1-transfected HeLa cells, the EBNA1 level was decreased to 42.5% and 17.6% for the treatments of curcumin at 5 *μ*M and 10 *μ*M, respectively (Figure 4(c)). These results were consistent with the results obtained from the cell apoptosis assay.

To further analyze whether the decreased EBNA1 expression level was induced by the alterations at the transcription level, quantitative real-time PCR was carried out for the HK1-EBV and HONE1 cells after drug administration. Our findings showed that compared with the control group, curcumin (5 *μ*M and 10 *μ*M) reduced the EBNA1 mRNA levels in the HONE1 cells to 70.12% and 39.52%, respectively. On the contrary, EBNA1 mRNA levels in HK1-EBV cells had been reduced to 60.18% and 37.26% for the 5 *μ*M and 10 *μ*M treatment groups, respectively (Figure 4(d)). The findings suggest that curcumin could inhibit the EBNA1 levels in the HK1-EBV and HONE1 cells.

### 3.5. Curcumin Decreases EBNA1 Promotor Qp Activities

EBNA1 transcription is initiated by Qp in EBV^+^ NPC cells. To determine the effects of curcumin on the EBNA1 promotor activity, the HeLa cells were transfected with pRL-TK, in combination with pGL3.0 or pGL3.0-Qp vector. After drug administration, the luciferase activity was measured. The Qp activities were reduced to 70.2% and 59.8% for the 5 *μ*M and 10 *μ*M curcumin treatment groups, respectively, (Figure 4(e)). Thus, curcumin treatment could decrease the transcriptional activity of EBNA1 in the HeLa cells.

### 3.6. Curcumin Reduces Stability and Promotes Proteasomal Degradation of EBNA1

To determine the effects of curcumin on the stability of EBNA1, the HONE1 and HK1-EBV cells were subjected to curcumin, under the condition of cycloheximide (CHX). Curcumin reduced the half-time of EBNA1 in HK1-EBV and HONE1 cells after CHX treatment compared with the control groups (Figures 4(f) and 4(g)).

In addition, the effects of curcumin on the proteasome pathway were investigated. HK1-EBV and HONE1 cells were incubated with curcumin, with or without the proteasome inhibitor (MG-132). MG-132 treatment increased the EBNA1 expression levels (Figures 4(h) and 4(i)) in both the HK1-EBV and HONE1 cells, either with or without curcumin. These findings suggest that curcumin could induce the EBNA1 degradation via the proteasome-ubiquitin pathway.

### 3.7. EBNA1 Overexpression Attenuates Effects of Curcumin

**Figure 5:**
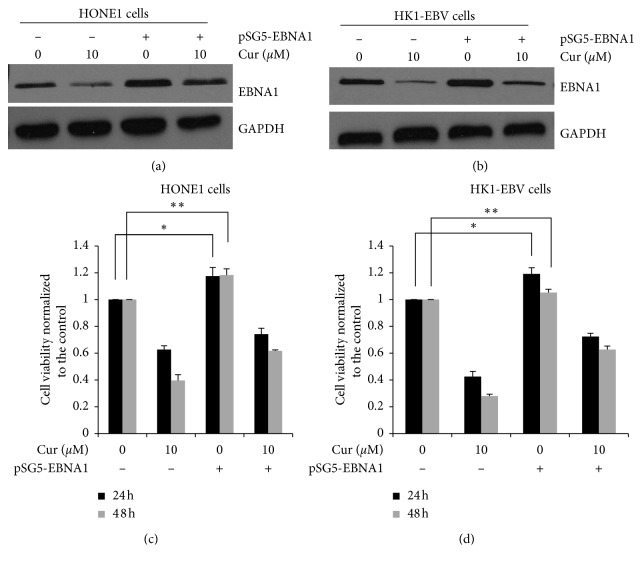
EBNA1 overexpression attenuates curcumin-induced cell killing and apoptosis-promoting effects in EBV-positive NPC cells. The HONE1 (a) and HK1-EBV (b) cells were transiently transfected with empty vector or pSG5-EBNA1 for 4 h followed by the treatment of curcumin (10 *μ*M). Western blot analysis was performed to detect the protein expression levels. Cell viabilities of HONE1 (c) and HK1-EBV (d) cells were determined by the CCK-8. Compared with the vehicle control (0.006% DMSO) group, ^*∗*^*P* < 0.05, ^*∗∗*^*P* < 0.01.

Whether the curcumin-decreased cell viability was induced by the inhibited EBNA1 expression was determined. The HK1 and HONE1 cells were transfected with pSG5-EBNA1 and then treated with curcumin. The transient transfection increased the EBNA1 expression levels in the HONE1 cells by 301.3% and in the HK1 cells by 368.1% (Figures 5(a) and 5(b)).

The cellular viability was then determined. As shown in Figures 5(c) and 5(d), curcumin treatment significantly decreased the cell viabilities of both HK1-EBV and HONE1 cells. After EBNA1 overexpression for 48 h, the cell viabilities of HONE1 and HK1-EBV cells were increased by 15.8% and 10.4%, respectively. The findings demonstrate that curcumin could inhibit EBV^+^ HONE1 and HK1-EBV cell proliferation, probably via decreasing EBNA1 expression.

### 3.8. Curcumin Inhibits Replication of EBV in NPC Cells Positive for EBV

**Figure 6:**
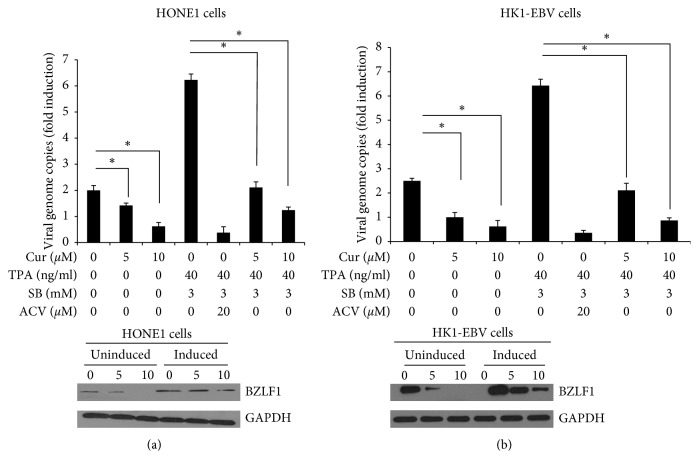
Curcumin reduces EBV virion production in EBV-positive NPC cells. The HONE1 (a) and HK1-EBV (b) cells were treated with TPA (40 ng/ml) and SB (3 mM) for 3 h followed by the treatment of DMSO (vehicle control) or curcumin (5 *μ*M and 10 *μ*M). The culture medium was collected and subjected to quantitative real-time PCR. Compared with the vehicle control (0.006% DMSO) group, ^*∗*^*P* < 0.05, ^*∗∗*^*P* < 0.01.

Effect of curcumin on the replication of EBV was determined. The TPA and NaB-induced HK1-EBV and HONE1 cells were treated with curcumin. Quantitative real-time PCR was performed to investigate the latent and lytic replication of EBV EBNA1 fragment. Curcumin treatment reduced the production of progeny virion, in a dose-dependent manner (Figures 6(a) and 6(b)). The findings demonstrate that curcumin could inhibit EBV latent and lytic replication in both HK1-EBV and HONE1 cells. Moreover, the effect of Curcumin on the EBV transcriptional activator BZLF1 encoded by IE was investigated with western blot analysis. Our results showed that curcumin significantly reduced the expression levels of BZLF1 in both induced and noninduced HONE1 and HK1-EBV cells.

## 4. Discussion

It has been demonstrated that the infection of EBV is closely linked to NPC [28]. EBNA1 could regulate the DNA synthesis of EBV, which contributes to the cellular proliferation, survival, tumorigenesis, and immortalization, which provides an attractive target for the EBV-related diseases [4, 29, 30]. Previous studies from our lab have shown that the Hsp90 inhibitor and berberine could inhibit the proliferation of malignant cells infected with EBV via the EBNA1-dependent pathway [13, 25]. It is clinically significant to find and develop the drugs that could inhibit the activity of EBNA1. Herein, our findings indicated that curcumin reduced the EBNA1 expression via the proteasome-ubiquitin pathway, both *in vivo* and *in vitro*. Moreover, EBNA1 overexpression could attenuate the curcumin-induced NPC cell apoptosis.

Curcumin is a rhizome extracted from the herb *Curcuma longa* Linn., which is frequently used in Ayurveda and traditional Chinese medicine to prevent and cure a variety of ailments in human beings [31, 32]. It has been shown that curcumin might be applied as efficient treatments for various disorders, including the allergy, coryza, sinusitis, hepatic diseases, cough, asthma, and bronchial hyperactivity [33–35]. Moreover, curcumin could exert the antioxidant, antiarthritic, hepatoprotective, thrombosuppressive, and cardioprotective effects [36, 37]. Furthermore, due to its distinct chemical properties, curcumin has also been reported as a potent anticancer compound [38, 39]. Curcumin could interact with various intracellular and extracellular molecules actively implied in the initiation as well as progression of cancers, thereby inhibiting the disease progression [40].

Increasing evidence has suggested that the inflammatory pathways play pivotal roles in a variety of chronic diseases, including cancers [41]. EBNA1 is a vital protein for the replication and persistence of EBV episomes [12, 42]. Herein, curcumin reduced the EBNA1 level in the EBV^+^ NPC cells and EBV^−^HeLa cells, which had pSG5-EBNA1 transfection. In addition, the EBNA1 stability was declined by curcumin treatment. Curcumin could inhibit the mRNA and protein levels of EBNA1 in the HK1 and HONE1 cells.

A previous study from our lab has indicated that triptolide could suppress the LMP1 promoter ED-L1 activity in the B lymphocytes positive for latency III type infected EBV [26]. In NPC, the EBV would expresse type II latency. Herein, the EBNA1 mRNA expression levels were decreased by curcumin treatment. Moreover, the EBNA1 promoter Qp activity was inhibited by the curcumin treatment, indicating that the decreased activity of the EBNA1 promoter would inhibit the transcription levels of EBNA1.

Previous studies have suggested that curcumin could activate caspase-3, caspase-8, and caspase-9 and PARP protein in various cancer cell lines [43, 44]. However, increasing evidence has demonstrated that the essential apoptotic pathways represent the mitochondria-dependent and death receptor-mediated pathways [45]. In this study, our results suggested that curcumin could induce EBV^+^ NPC cell apoptosis, through the caspase-9-involved and death receptor-mediated pathways.

In addition to the EBV-encoded protein latent membrane protein 1 (LMP1), EBNA1 also regulates the viral transcription and exerts multiple functions on cellular proteins, as well as on the pathways promoting cell survival and proliferation [14]. A previous study from our lab has shown that the LMP1 overexpression would increase the EBV^+^ B lymphocyte (B95-8 and P3HR) viabilities [26]. Therefore, we speculate whether the EBNA1 overexpression could increase the viabilities of HK1-EBV and HONE1 cells. Our findings showed that EBNA1 overexpression attenuated the inhibitory effects of curcumin. Moreover, the curcumin treatment reduced the EBV^+^ NPC cell proliferation and activities, at least partially, related to the reduced EBNA1 level.

## 5. Conclusions

In conclusion, our findings indicate that curcumin reduced cell proliferation and apoptosis and changed cycle arrest in the EBV^+^ NPC cells. Moreover, the decreased Qp promoter activity provides evidence for curcumin-induced declined EBNA1 mRNA levels. In addition, EBNA1 overexpression attenuates the effects of curcumin in EBV^+^ NPC cells. Based on these findings, curcumin might make a novel therapy for a variety of tumors in clinics, including NPC. Finally, although our results suggested that curcumin might reduce the total number of EBV-positive cells *in vitro*, the *in vivo* test should be carried out, and the long-term toxicity of curcumin should be estimated. Moreover, the safety and efficacy of curcumin would need to be investigated in clinical trials.

## Data Availability

The data used to support the findings of this study are available from the corresponding author upon request.
